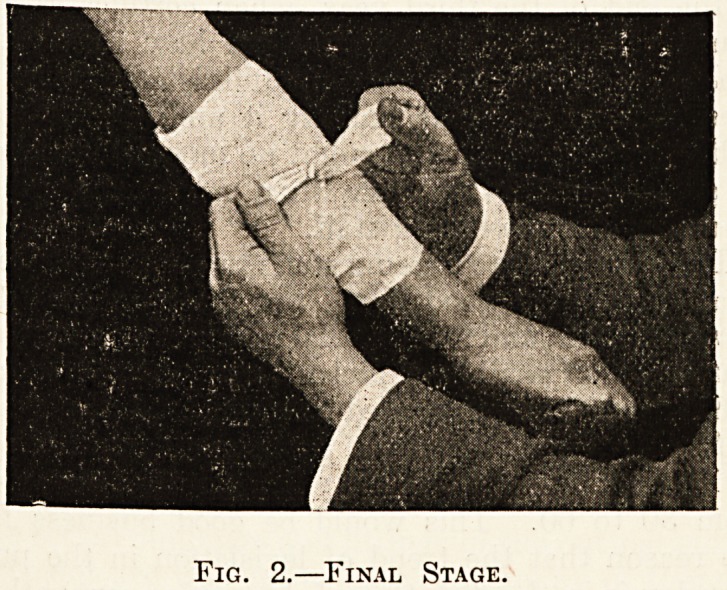# New Appliances and Things Medical

**Published:** 1909-11-20

**Authors:** 


### NEW APPLIANCES AND THINGS MEDICAL.

We shall be glad to receive at our Office, 28 & 29 Southampton Street, Strand, London, W.C., from the manufacturers, specimens of all new preparations and appliances.]

### HYOSCINE-MORPHINE-CACTIN COMPOUND (H. M. C. ABBOTT)

(E. J. REID AND CO., 17 and 18 BASINGHALL STREET, LONDON, E.C.).

WE have received a sample of the above hypodermic tablets. These are intended for subcutaneous use for the production of general anæsthesia. Each contains $\frac{1}{100}$th of a grain of hyoscine hydro bromide, $\frac{1}{4}$ of a grain of morphine hydro bromide, and $\frac{1}{67}$th of a grain of cactine. A weaker tablet, of which we have also received a sample, contains one-half the above quantities. We cannot enter here into the use and efficacy of hypodermic injections of the above kind for the production of general anæsthesia sufficiently deep for surgical purposes. They may be used either alone or followed by chloroform or ether. There is no doubt that general anæsthesia more or less deep follows about an hour after such injections. The combination of drugs which has been most frequently employed up to the present has been a combination known as scopolamin-morphine, and it appears during 1908 nearly 24,000 general anæsthesias have been thus produced in the large German hospitals. Passing for the moment over the question as to the identity of scopolamin and hyoscine, the introduction of cactin into the present product distinguishes it from all others. There is no doubt from the literature that these tablets will produce the effects expected of them. We should suggest, however, to those who wish to try them that the weaker tablet should be used first, and that if sufficiently deep sleep does not ensue within an hour that the dose should be repeated.

### AN IDEAL EMERGENCY BANDAGE.

ASEPTO EMERGENCY BANDAGE. London Agents. Mr. John Voet, 2 Coleman Street, E.C.

WE have received several samples of this simple and exceedingly interesting invention. The "Asepto Emergency Bandage" consists of a pad of wadding enclosed in bandage cloth, to which is attached an ordinary roll bandage. The whole is folded up, and when put up in its casing or

FIG. 1.—FOLDED PAD IN POSITION.

oiled paper, is very compact, so much so in fact that three of the little packets can be contained in the palm of the hand, each packet weighing only a few grams. But the bandage is something more than a compact emergency bandage; it is an absolutely sterile contrivance, and the pad is so folded that it can be thrown about, and its outside dirtied without becoming contaminated on the inside. We have opened one of the little packages and allowed it to drop on the street, without completely unwinding the bandage that protects the central pad, and then taken cultivations from the centre of the pad, and have found this centre absolutely sterile. We have also tested the bandage in practice, and found it thoroughly satisfactory as a covering for wounds. At a pinch the pad serves as an excellent surgical dressing. The accompanying blocks give an idea of the general way in which the bandage is applied. The beauty of this little bandage can scarcely be adequately realised by those who have not handled it; it needs to be seen to be properly appreciated. To the hospital surgeon who has at his command fully sterilised bandages and cotton wool, it is of course not so valuable as it is to the ambulance man, to the soldier, or to anyone who

FIG. 2.—FINAL STAGE.

is called upon to put on an emergency bandage. To such it should prove a great boon, for it realises the ideal in the way of bandages. It is put up in so compact and so absolutely sterile a form in oiled paper packets, or for harder wear and the use of those who are exposed to all weathers, with an outer cover of waterproof cloth, that it is always available and ready for use. The bandage, we understand, has been adopted by the ambulance service of the various Continental armies and railways by the St. John's Ambulance Association, and by various city ambulances. To all ambulance services that do not already possess it it can be cordially commended, and also to travellers, tourists, and, indeed, to everyone who may at any time stand in need of a thoroughly reliable emergency bandage. There is no other type on the market, so far as we know, that can compete with the Asepto, either in simplicity, reliability, compactness, or general excellence. Samples and full particulars as to price, etc., may be obtained on application to Mr. John Voet, 2 Coleman Street, E.C.

THE fourth edition, dated October 1909, of the "Book of Scotch-made Underwear" has just been issued by Greensmith Downes and Son, the well-known Edinburgh makers of woollen hosiery. The book is a careful compilation of facts relating to the hygienic value of wool as a substance to be worn next the skin, and forms an attractive advertisement of the amazing variety of goods manufactured by the firm. For not undergarments only, but every conceivable part of clothing that can be made advantageously of wool for women and men, is mentioned and illustrated. There are several coloured illustrations and patterns of wool stuffs in the book.

## Figures and Tables

**Fig. 1. f1:**
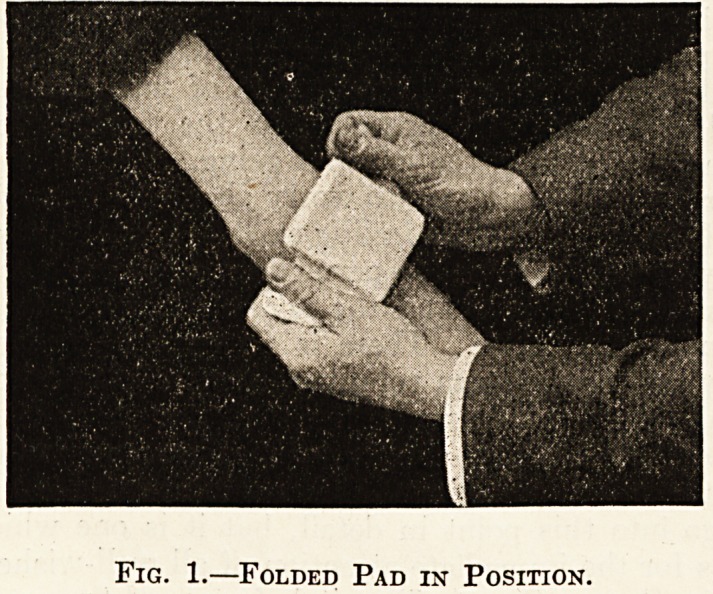


**Fig. 2. f2:**